# Oxidation state and metasomatism of the lithospheric mantle beneath the Rae Craton, Canada: strong gradients reflect craton formation and evolution

**DOI:** 10.1038/s41598-021-83261-6

**Published:** 2021-02-11

**Authors:** Alan B. Woodland, Carolin Gräf, Theresa Sandner, Heidi E. Höfer, Hans-Michael Seitz, D. Graham Pearson, Bruce A. Kjarsgaard

**Affiliations:** 1 Institut Für Geowissenschaften, Goethe-Universität Frankfurt, Altenhöferallee 1, 60438 Frankfurt am Main, Germany; 2 Frankfurt Isotope and Element Research Center (FIERCE), Goethe-Universität Frankfurt, Frankfurt am Main, Germany; 3 Department of Earth and Atmospheric Sciences, University of Alberta, 1-26 Earth Science Building, Edmonton, AB T6G 2E3 Canada; 4 Geological Survey of Canada, 615 Booth Street, Ottawa, ON K1A 0E9 Canada

**Keywords:** Solid Earth sciences, Geochemistry, Petrology

## Abstract

We present the first oxidation state measurements for the subcontinental lithospheric mantle (SCLM) beneath the Rae craton, northern Canada, one of the largest components of the Canadian shield. In combination with major and trace element compositions for garnet and clinopyroxene, we assess the relationship between oxidation state and metasomatic overprinting. The sample suite comprises peridotite xenoliths from the central part (Pelly Bay) and the craton margin (Somerset Island) providing insights into lateral and vertical variations in lithospheric character. Our suite contains spinel, garnet-spinel and garnet peridotites, with most samples originating from 100 to 140 km depth. Within this narrow depth range we observe strong chemical gradients, including variations in oxygen fugacity (ƒO_2_) of over 4 log units. Both Pelly Bay and Somerset Island peridotites reveal a change in metasomatic type with depth. Observed geochemical systematics and textural evidence support the notion that Rae SCLM developed through amalgamation of different local domains, establishing chemical gradients from the start. These gradients were subsequently modified by migrating melts that drove further development of different types of metasomatic overprinting and variable oxidation at a range of length scales. This oxidation already apparent at ~ 100 km depth could have locally destabilised any pre-existing diamond or graphite.

## Introduction

Cratons contain the oldest rocks on Earth, exceeding 2 Ga in age, and have played an integral role in the development of the continental crust^1,2^. One factor allowing the long-term (> 1 Ga) preservation of such crustal rocks is the existence of a thick lithospheric root that may extend to 250 km depth or more and which is chemically and physically distinct from the surrounding asthenosphere^3–6^. As such, the formation and physico-chemical evolution of this thick SCLM is integral to our understanding of crust-mantle dynamics from the early Earth to the present.

**Figure 1:**
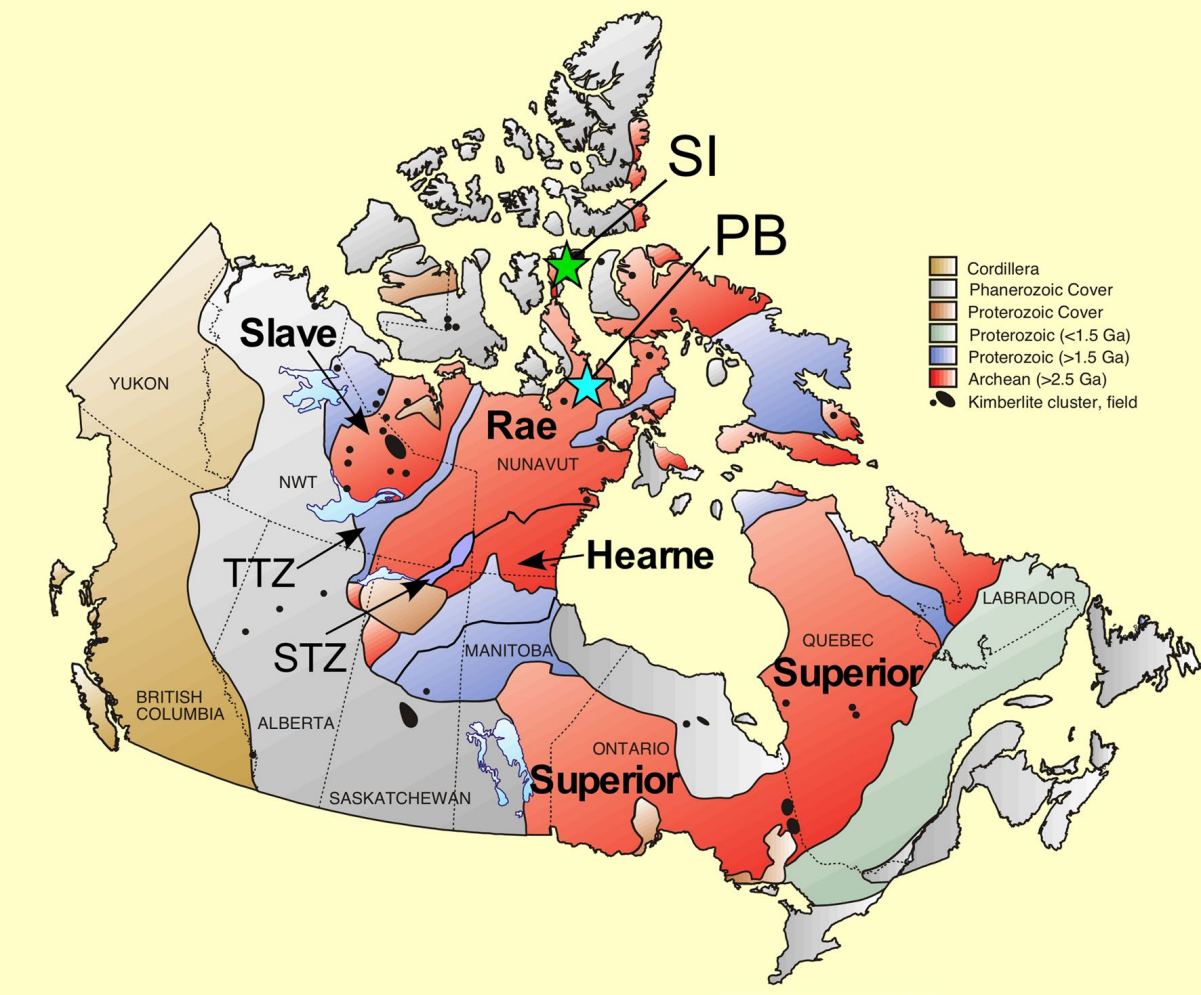
Schematic map of the Canadian shield illustrating the peridotite xenolith localities along with the constituent cratonic blocks and surrounding tectonic zones. *SI* Somerset Island, *PB* Pelly Bay, *TTZ* Thelon–Taltson magmatic zone, *STZ* Snowbird tectonic zone (modified after Kjarsgaard^13^).

The Rae craton is one of the large Meso-Neoarchean cratons that make up the Canadian Shield (Fig. 1). It comprises a number of component crustal blocks that were amalgamated in the Archean at ca. 2.7–2.6 Ga^6–9^. Somerset Island is located in the northernmost part of the Queen Maud block and Pelly Bay is situated on the Committee block, in the north-central Rae (Fig. 1). The Rae craton is bounded to the west by the 1.97 Ga Thelon-Taltson magmatic zone, which developed during collision with the Slave craton^7,10^. Likewise, the Hearne craton accreted onto the southern margin of the Rae at 1.92 Ga, as presently represented by the Snowbird tectonic zone^10–12^.

Radiogenic isotope studies of peridotite xenoliths erupted by kimberlites on Somerset Island^14–19^ revealed that the cratonic lithosphere stabilised by at least 2.8 Ga. Rhenium depletion model ages yield a broad mode in their distribution from 3.0 to 2.25 Ga, with a mean of 2.3 ± 0.3 Ga, with no discernable relationship between age and depth^18^. In contrast, depletion model ages for peridotite xenoliths from Pelly Bay produce three age groups: Archean 2.8–2.6 Ga, Paleoproterozoic 2.1–1.7 Ga and “Recent” < 1 Ga^9^. The Paleoproterozoic peridotites tend to occur at deeper levels, suggestive of a chronologically and structurally layered lithosphere, and are thought to represent more juvenile mantle, possibly related to metasomatic and/or magmatic interactions from regional-scale underplating during the Kivalliq–Nueltin event at 1.77–1.7 Ga^9^.

Despite the large size of the Rae craton, much less is known about its mantle root than the neighboring small Slave craton, although recent geophysical studies have provided important information on its lithospheric structure^6,8,20^. However, no data are currently available on the oxidation state of the Rae SCLM. We have determined the oxidation state of two suites of peridotite xenoliths, one from the interior of the craton at Pelly Bay and one from the craton margin at Somerset Island. These results are then discussed within the context of differing metasomatic signatures recorded by garnet and clinopyroxene and depth of origin, as assessed by geothermobarometric calculations.

## Results

### Peridotite xenolith samples

A total of 30 peridotite xenoliths were investigated: 25 from four different kimberlite localities (Batty Bay, Nikos, JP north, JP south) on Somerset Island and five samples from Pelly Bay. Somerset Island peridotites were previously studied by Irvine et al.^18^ and Bragagni et al.^19^ primarily for whole-rock Re-Os and PGE geochemistry and comprise five spinel peridotites, ten garnet peridotites and ten garnet-spinel peridotites. In the case of the garnet-spinel peridotites, the material available to us for five of the samples only contained spinel (i.e. no garnet). The Somerset Island kimberlites were emplaced 93–98 Ma ago^21^. The smaller suite from Pelly Bay consists of one spinel lherzolite and two garnet-spinel lherzolites and two garnet lherzolites and is a subset of samples investigated by Liu et al.^9^ where they also focused on PGE geochemistry and Re-Os dating. Kimberlite emplacement at Pelly Bay has been dated at 546 Ma^22^.

The Somerset Island samples mostly exhibit coarse equant or coarse tabular textures with only four samples (N1C, N2B, K11-A18, K13-A3) being porphyroclastic^23^, following the nomenclature of Harte^24^ (Supplementary Fig. S1). The stronger alteration of the Pelly Bay xenoliths precludes a textural assessment for several of the samples, but TU13-A2-3 and TU14-B2 are coarse tabular and porphyroclastic, respectively. Kelyphite alteration rims are variably developed around the garnets. Spinel is generally interstitial to olivine and ranges in color from brown to red-brown to dark brown, reflecting its Cr/Al ratio (see below). In several cases, spinel occurs with a vermicular texture with clinopyroxene, orthopyroxene ± olivine (Supplementary Fig. S1), suggesting that these intergrowths formed by the breakdown of garnet during upwelling^25^. In many garnet-spinel peridotites the two phases are not spatially associated with each other, suggesting that spinel and garnet may not necessarily be in mutual equilibrium (Supplementary Fig. S1).

### Major element mineral chemistry

Major element compositions of analysed minerals are presented in Supplementary Tables 1, 2, 3, 4 and 5. The Fe^3+^/∑Fe of garnet, determined either by Mössbauer spectroscopy or the flank method (see “Methods”), is also reported in Supplementary Table 4. The Fe^3+^/∑Fe of spinel, determined either by Mössbauer spectroscopy or using secondary standards (see “Methods”) is likewise listed in Supplementary Table 5. The minerals have compositions similar to those reported for upper-mantle xenoliths from other worldwide localities. Olivine Mg# values (Mg# = 100 × Mg/(Mg + Fe)) vary from 90.8 to 92.8 and NiO contents range from 0.31 to 0.43 wt%, with most > 0.37 wt%. Orthopyroxenes have Mg# values ranging from 91.7 to 93.5, Al_2_O_3_ contents of 0.88–3.2 wt% and CaO contents up to 0.87 wt% with an average of 0.57 wt%. Where present, clinopyroxene is Al-rich Cr-diopside with higher Mg# and Al_2_O_3_ contents in the spinel peridotites compared to those in the garnet-bearing samples (Fig. S2).

In a Cr_2_O_3_–CaO classification diagram all garnets plot within the G9, lherzolitic, field defined by Grütter et al.^26^ (Fig. S3). Garnets from Pelly Bay are richer in Fe (ave. Mg# = 78) than those from Somerset Island (ave. Mg# = 85), but Cr contents overlap between the two suites (Supplementary Fig. S3). Fe^3+^/∑Fe ranges from 0.03 to 0.07 in the Pelly Bay garnets and from 0.03 to 0.14 in those from Somerset Island. Spinel compositions form two broad clusters in terms of their Mg# and Cr#; one group having Mg# < 63 and Cr# ≥ 70 and the other group with Mg# ≥ 65 and Cr# < 42 (Supplementary Fig. S4). Except for one spinel harzburgite (JP3-X1), all other Cr-rich spinels are from garnet-spinel peridotites. Two other garnet-spinel peridotites contain spinels with relatively low Cr contents (K11-A18, K15-A4, Supplementary Fig. S4). Spinels have Fe^3+^/∑Fe ranging from 0.01 to 0.26 (Supplementary Table 5).

### Trace element mineral chemistry

**Figure 2:**
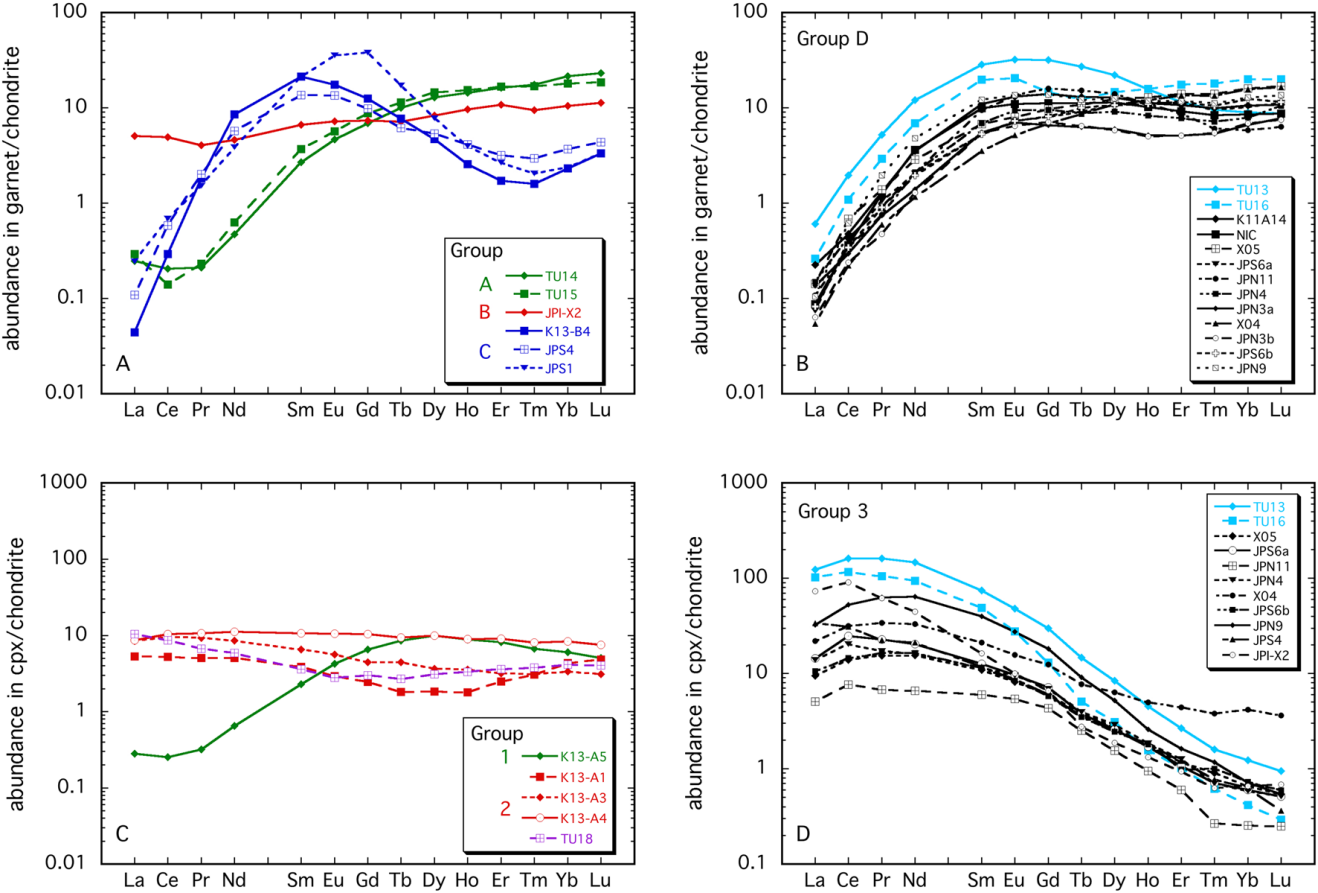
Chondrite-normalized REE patterns for garnet (**A**,**B**) and clinopyroxene (**C**,**D**) illustrating the different signatures as described in the text. Normalization is after McDonough and Sun^27^. The samples in blue in **B**–**D** are from Pelly Bay, the rest being from Somerset Island.

Trace element abundances in garnet and clinopyroxene determined by LA-ICP-MS (see “Methods”) are presented in Supplementary Table 6. The chondrite-normalised^27^ Rare Earth Element (REE) signatures of garnet permit the samples to be divided into four groups (Fig. 2A,B). Two samples from Pelly Bay (group A) exhibit a slight negative slope between La_N_ and Ce_N_, followed by steadily increasing normalised concentrations of light, middle and heavy Rare Earths (LREE_N_, MREE_N_ and HREE_N_, respectively). Although these patterns have the appearance of being geochemically “depleted”, they have some of the highest concentrations of HREE and other elements like Y and Ga that are recognised as indicators of enrichment processes^28^ (Fig. 2A, Supplementary Table 6). Garnet in one sample from Somerset Island has a flat normalised pattern (Group B, Fig. 2B). Group C samples have “sinusoidal” patterns with a peak occurring for the MREE_N_ that systematically record the some of the lowest Ti, Yb, Y and Ga abundances in the sample set, reflecting a relatively depleted character^28^ (Fig. 2A, Supplementary Table 6), though the elevation of La and Ce over the MREE indicates some degree of enrichment. The majority of the xenoliths contain garnet exhibiting “normal” patterns^29^ with normalised abundances rising steeply from La_N_ and then either remaining flat or having a slight sinusoidal character from Sm_N_ to Lu_N_ (Fig. 2B). These are designated as group D, with two samples from Pelly Bay having the highest MREE_N_ and LREE_N_ concentrations.

Trace element signatures of clinopyroxene can be divided into three groups. One spinel peridotite has a pattern with depleted LREE_N_ and a hump-shaped form across the MREE_N_ and HREE_N_ with a maximum at Dy_N_ (Fig. 2C). Clinopyroxenes in four other spinel peridotites have fairly flat REE_N_ patterns, with slightly elevated LREE_N_ abundances (Fig. 2C). Yb concentrations are also quite high in these samples (> 0.5 µg/g, Supplementary Table 6). The remaining samples that contained analysable clinopyroxene exhibit enriched patterns with abundances steadily decreasing from Ce_N_ to Lu_N_ with a general flattening off between La_N_ and Nd_N_ (Fig. 2D). These samples are all garnet-bearing and in most cases, the coexisting garnet has a “normal” REE, i.e. group D pattern (two exceptions are JP1-X2 and JPS4, where the garnet has a flat or sinusoidal normalised REE signature, respectively).

### Thermobarometry

The Somerset Island samples record equilibration temperatures from 830 to 1150 °C and pressures of 3.1–4.6 GPa, while the limited number of peritotites from Pelly Bay yield temperatures of 674–905 °C with pressures ranging from 2.5 to 4.0 GPa (see “Methods” and Supplementary Table 7). This corresponds to a depth range of approximately 100–150 km at Somerset Island and from ~ 80 to 130 km depth for the Pelly Bay suite. Thus our samples cover a depth range of ~ 50 km through the lithospheric mantle at both localities.

### Oxygen fugacity measurements

The *ƒ*O_2_ values for garnet-bearing peridotites were calculated after Stagno et al*.*^30^ and for spinel-bearing peridotites after the Nell-Wood model^31^ (see “Methods”). Garnet-bearing peridotite xenoliths from Pelly Bay have Δlog*ƒO*_2_ values between FMQ − 1.99 and FMQ − 1.35 (Supplementary Table 7). The only garnet-free xenolith from this suite (TU18) has a very low oxidation state of Δlog*ƒO*_2_ = FMQ − 4.13. The peridotites from Somerset Island record Δlog*ƒO*_2_ values between FMQ − 3.61 and FMQ + 1.08. For three garnet-spinel peridotites from Somerset Island and two from Pelly Bay, ∆logƒO_2_ values could be estimated on the basis of Fe^3+^ contents in both garnet and spinel. In one case (NIC), values overlap within error and in the other cases (JPS, JPN9, TU13, TU16) the spinel-based equilibrium yielded values significantly more oxidised, underlining the fact that spinel and garnet may not always be in equilibrium with eachother (Supplementary Table 7). In fact these two phases are not generally observed to be in physical contact in our samples (e.g. Supplementary Fig. S1) and this behavior suggests that garnet Fe^3+^/∑Fe may be sluggish to reset to changing redox conditions. This would be consistent with the fact that Fe^3+^ and Fe^2+^ reside on different crystallographic sites in garnet so that a change in Fe^3+^ content requires cation exchange (by diffusion or recrystallisation) rather than just the migration of an electron. Additionally, it could be related to the complex process of redistributing ferric iron between phases at the garnet-spinel transition since this also involves changes in the compositions and modes of orthopyroxene and clinopyroxene, along with the exchange of Fe^3+^ between garnet and spinel.

## Discussion

**Figure 3:**
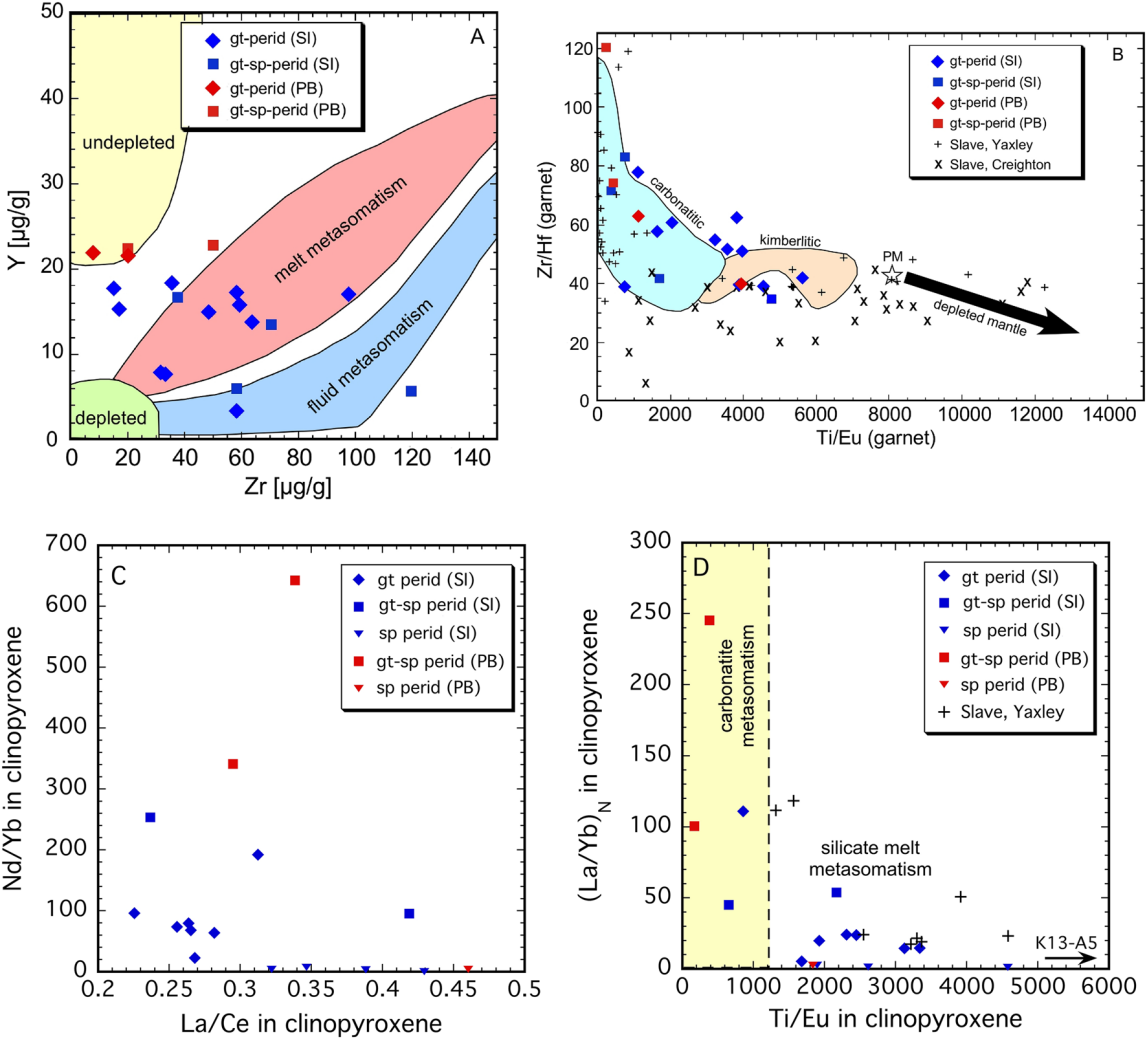
Selected trace element variations in garnet (**A**,**B**) and clinopyroxene (**C**,**D**) that can be used as indicators of metasomatic processes. Fields in (**A**,**B**) after Griffin et al.^32^ and Shu and Brey^33^, respectively. The demarcation of carbonatite and silicate melt metasomatism in (**D**) is from Coltorti et al.^35^.

The different REE signatures of garnet and clinopyroxene are process related; metasomatic interactions within the SCLM beneath the Rae craton can be further characterised and discriminated by considering further minor and trace element variations. The Zr–Y systematics of garnet (Fig. 3A) reveal that the three Somerset Island samples with sinusoidal REE patterns (group C) plot within the fluid-metasomatism field of Griffin et al.^32^ and their low Ti/Eu and variable Zr/Hf point to a carbonatitic metasomatic agent^33^ (Fig. 3B). Many of the group D garnets plot within the melt metasomatism field of Griffin et al.^32^ and are distributed across the fields of carbonatitic and kimberlitic metasomatism in terms of their Ti/Eu and Zr/Hf ratios (Fig. 3A,B). The group A and D garnets from Pelly Bay all have elevated Y contents and their Ti/Eu-Zr/Hf systematics place three of them in the field of carbonatitic metasomatism, with one group A sample (TU15) being significantly richer in Ti, suggesting interaction with a kimberlitic melt. Several samples from Somerset Island also exhibit similar Y enrichment with relatively no concomitant Zr enrichment (Fig. 3A), reminiscent of MARID-style metasomatism^34^. Thus, the portion of the Rae SCLM lying at 100–150 km records highly variable metasomatic interactions with at least three types of agent, carbonatitic, MARID and mafic silicate or kimberlitic melts; the geochemical signature of the mafic silicate melt being different between the craton margin at Somerset Island and mid-craton at Pelly Bay. Overall, the extent of Y- (and Ga) enrichment is similar to that observed for garnets from the deeper portions of the central Slave craton, the difference being that enrichment of the Rae SCLM occurs at significantly shallower depths (~ 50 km shallower; Supplementary Fig. S5). Our observed range in Ti/Eu for garnet is much less than reported for the Slave SCLM, all giving values < 6000 (Fig. 3B).

The trace element contents of clinopyroxene are consistent with this picture of several different types of metasomatic agents having interacted to different degrees with the peridotites. Diopside from the spinel peridotites in our suite generally exhibit the least extent of enrichment, as suggested by very low Nd/Yb and (La/Yb)_N_ ratios (Fig. 3C,D). However, sample K13-A5 stands out in terms of its extremely high Ti/Eu (Supplementary Table 6), reflecting a different type of metasomatism that caused Ti enrichment that also led to rutile formation. In contrast, clinopyroxene in four other spinel peridotites, including the shallowest-lying sample, TU18, has a Ti/Eu ratio distinctly lower than that expected for a primitive mantle assemblage (Ti/Eu = 4742, Uenver-Thiele et al.^36^), implying some degree of interaction with mafic silicate melts. Of the garnet-bearing samples, four have clinopyroxene with Ti/Eu < 1200, which is indicative of carbonatitic metasomatism^35^ (Fig. 3D). Garnet compositions from these samples are consistent with this interpretation, including two samples from Pelly Bay (compare Fig. 3B,D). In fact, comparison of calculated partition coefficients between garnet and clinopyroxene (D^gt/cpx^) are indicative of trace element equilibrium as defined by Zack et al.^37^ in the samples where both phases could be analysed (see “Supplementary Information S1”, Supplementary Fig. S6).

**Figure 4:**
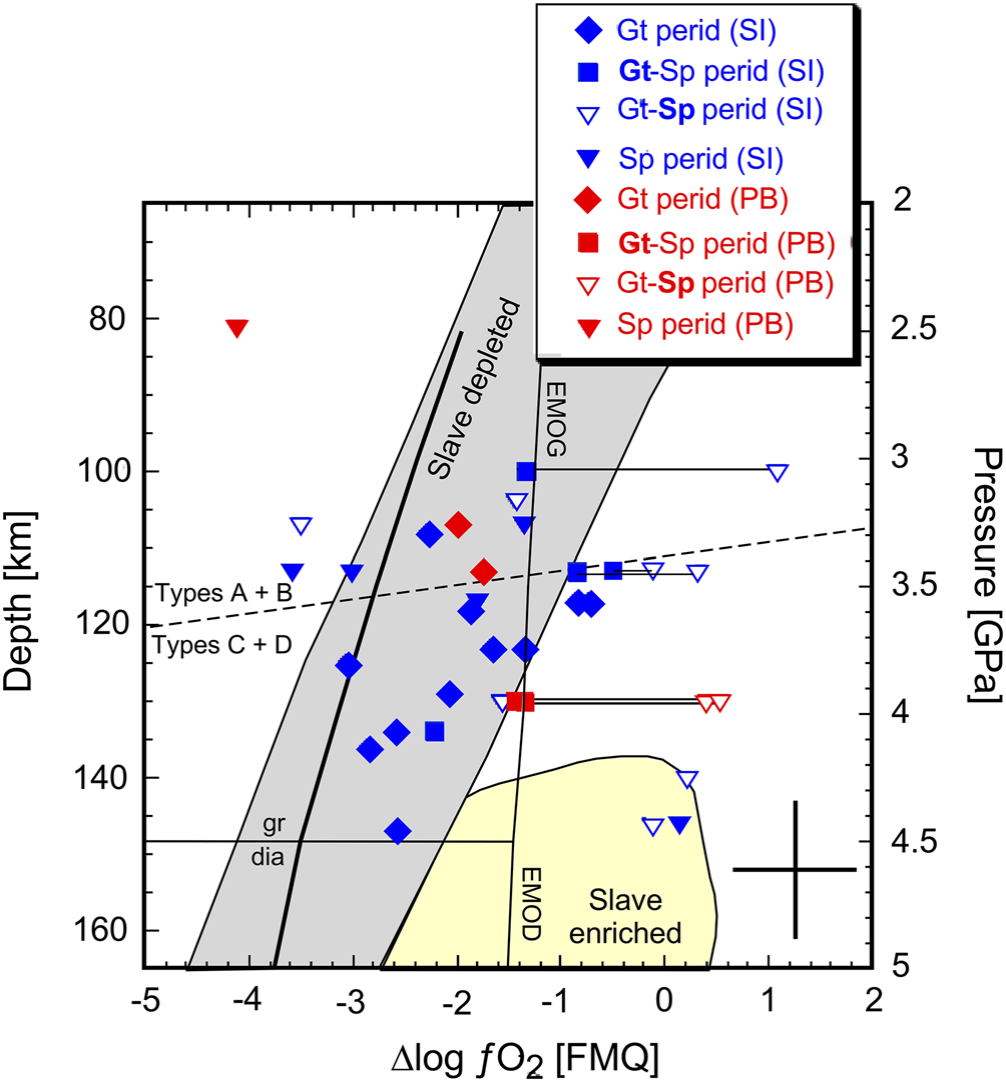
Variation in ∆logƒO_2_ as a function of pressure/depth for samples from this study. Pressure was converted to depth assuming Depth (km) = P (GPa)*32.4. Thin horizontal lines connect ƒO_2_ values determined from garnet (solid) and spinel (open) equilibria from the same garnet-spinel peridotite xenolith. The dashed line demarcates the occurrences of type A and B garnets with respect to those with type C and D signatures. The grey and yellow fields encompass the depleted and enriched samples reported for the Slave craton (Yaxley et al.^41^; Creighton et al.^42^). The thick solid line illustrates the predicted variation for pre-metasomatic (primitive) mantle peridotite along a 40 mW/m^2^ geotherm from Luth and Stachel^38^. The position of the graphite-diamond (gr-dia) transition was adopted from Kennedy and Kennedy^43^. EMOG and EMOD represent the positions of equilibria controlling carbonate stability in harzburgitic bulk compositions: enstatite + magnesite = forsterite + graphite (G) or diamond (D) + O_2_ from Luth and Stachel^38^, computed for a 40 mW/m^2^ geotherm. The black cross in the lower right hand corner illustrates the estimated uncertainties in the ƒO_2_ and pressure calculations.

Variations in oxidation state range up to ~ 4 log units in our suites from Pelly Bay and Somerset Island (Fig. 4, Supplementary Table 7). This provides evidence for strong redox gradients present at mid-lithosphere depths. Along with the shallowest sample, a spinel peridotite from Pelly Bay (TU18), several deeper-lying spinel-bearing peridotites from Somerset Island also record low ∆logƒO_2_ values of ≤ FMQ-3, even when considered relative to the predicted depth-ƒO_2_ curve of Luth and Stachel^38^ for a primitive mantle bulk composition with Fe^3+^/∑Fe = 0.02^39^ that we take to represent pre-metasomatic cratonic lithosphere (Fig. 4). However, the relatively low Ti/Eu and flat REE signatures of clinopyroxene in these samples attest to a certain degree of metasomatism that was apparently not significantly oxidising since the oxidation state of spinel peridotites can otherwise be readily reset by minor interactions^40^. Most garnet-bearing peridotites record higher ∆logƒO_2_ values compared to the estimated primitive mantle systematics of Luth and Stachel^38^, consistent with them having experienced a variety of metasomatic interactions, as described previously (Fig. 4). The ∆logƒO_2_ values derived from Fe^3+^/∑Fe in spinel in some garnet-spinel peridotites are always higher than values determined from corresponding garnet Fe^3+^ contents, implying a late oxidative event during which the garnet composition was only partially reset. It is noteworthy that such oxidising conditions will also destabilise any pre-existing graphite or diamond (Fig. 4).

The range on oxidation state we observe for Rae SCLM is significantly greater than that reported for the mantle section beneath Udachnaya^44^ and is comparable with similarly metasomatised peridotite xenoliths from Kimberley^45^. The relatively oxidised domains occur at significantly shallower depths beneath the Rae craton compared to that observed for the Slave SCLM^41,42^ (Fig. 4). This mirrors the aforementioned enrichment of certain metasomatic indicator elements like Y and Ga also at shallower depths than reported for the Slave SCLM (Supplementary Fig. S5).

An important observation is that our peridotite suites comprise different mineralogies (spinel, garnet-spinel and garnet peridotites) in spite of the fact that the thermobarometric calculations reveal that virtually all samples originated from a fairly narrow depth range of 100–150 km (Supplementary Table 7). The observed variations in the Yb content of garnet (Supplementary Table 6) point to different mantle domains having experienced differing degrees of depletion by partial melting prior to being juxtaposed within the cratonic root (e.g. Creighton et al.^42^). In addition, the relatively high Cr# of some garnets (> 0.2) suggests an earlier history of partial melting at shallower depths, within the spinel stability field^46,47^. On the other hand, the occurrence of vermicular spinel in some samples, particularly sample TU18, suggest vertical movements from the garnet into the spinel stability field have also taken place. The physical separation of garnet and spinel in the garnet-spinel peridotites also implies multiple stages of mineral formation during and after consolidation of the lithospheric root beneath the Rae craton. On a regional scale, the SCLM beneath Pelly Bay is noticeably richer in FeO compared to the craton margin at Somerset Island (Supplementary Tables 2, 3 and 4) and differences in trace element signatures are also apparent between the two localities (Figs. 2A–D, 3 and Supplementary Fig. S5). These observations appear to be consistent with the numerical models of Wang et al*.*^48^ that envision a two-stage process of craton development with initial tectonic shortening followed by a subsequent phase of gravitational self-thickening. Oscillatory instabilities within the root can develop due to relative buoyancy, but can be attenuated by secular cooling that promotes stabilisation of the thickened lithosphere. Such a model would involve significant lateral and vertical movement, helping to explain the juxtaposition of different mantle domains at similar depths. This also implies that redox gradients at different scales will be the rule rather than the exception in cratonic mantle, promoting the possibility of redox melting as a process for generating small volume melts (e.g. Foley^49^).

The metasomatic signature is observed to change as a function of depth, with garnet groups A and B lying at shallow levels above ~ 115 km and the more strongly enriched garnets belonging to groups C and D are found deeper (Fig. 4). An exception is one garnet-spinel peridotite (JPN9) that came from a depth of 100 km and is one of the most enriched samples of our suite (Fig. 2B,D). This distribution points to at least some metasomatic interactions having occurred since amalgamation of the cratonic root, with the agents rising from the asthenosphere. The picture is similar to that portrayed by Snyder et al*.*^6,20^ in their interpretive cross-section through the Rae craton, based in part on conductivity data. In this scheme, the SCLM peridotite domain represented by JPN9 would represent a finger of metasomatising melt that penetrated to shallower depths and caused partial oxidation (> FMQ) as recorded by the spinel. This contrasts with several spinel peridotites that originated from similar depths, but did not experience any oxidation. Thus the observed variations in mineralogy, metasomatic signatures and oxidation state of the SCLM at 100–150 km provide clues to the formation and evolution of the Rae craton in which tectonic juxtaposition of discrete mantle domains are an important feature of the construction of cratons. Not only does the assembly of the cratonic root produce geochemical gradients, but subsequent migration of melts through the consolidated lithospheric mantle cause additional heterogeneities to develop at different length scales, including locally modifying the oxidation state and influencing diamond stability.

## Methods

### Electronprobe microanalysis

Major and minor element analyses of minerals were determined with a JEOL JXA-8900 superprobe at Goethe Universität Frankfurt am Main. Measurements were made in wavelength dispersive mode with an acceleration potential of 15 kV, a beam current of 20 nA, a spot diameter of 3 µm and employing a mix of natural and synthetic standards for calibration. Depending on the analysed element, counting times on the peak were 15–40 s and 20–40 s on the background. Representative mineral compositions are presented in Supplementary Tables 1, 2, 3, 4 and 5.

### Laser-ablation-ICP-MS

Trace element abundances in garnet and clinopyroxene were analysed in situ with a single collector sector field ICP-MS system (Element 2, ThermoFisher Scientific) combined with a Resolution M-50 (Resonatics) 193 nm ArF excimer laser (ComPexPro 102F, Coherent) system at the Frankfurt Isotope and Element Research Center (FIERCE) Goethe Universität Frankfurt am Main. Ablation was done in a 0.45 l/min He stream and with an Ar sample gas flow rate of 0.85 l/min. RF Power was set to 1350 W resulting in an oxide production rate of 0.5–0.6%. Spotsizes of 26 µm and 40 µm were employed for clinopyroxene and garnet, respectively. The pulse frequency of the laser was set to 15 Hz and a laser energy of 100 mJ at 50% attenuation was applied, resulting in a laser fluence of 6–7 J/cm^2^. Measurements were performed in low-resolution mode with 60 s acquisition times (20 s for background and 40 s for the measurement). NIST 612 glass was used for external calibration, with BIR-1 (USGS) glass and an in-house garnet standard PN2b serving as secondary standards. As an internal standard we used the Si content measured by microprobe from the same area of the particular grains. Average element concentrations obtained for the standards and for selected elements are given in Supplementary Table 6. The standard error obtained by comparison of measured and published average element concentrations for BIR-1 glass is around ± 10%. Data reduction was carried out on-line with the GLITTER software^50^.

### Determination of Fe^3+^/∑Fe in garnet and spinel

Accurate measurement of Fe^3+^ concentrations in garnet or spinel is essential for estimating the oxidation state of the peridotites. Where adequate sample material was available, Fe^3+^/∑Fe was determined by Mössbauer spectroscopy at the Goethe Universität Frankfurt am Main following the data collection and analysis procedures described in Woodland & Koch^51^ and Woodland et al.^40^ for garnet and spinel, respectively. Optically clean separates were hand-picked under a binocular microscope. Spinels were treated sequentially with HF and HCl to eliminate any adhering silicates or magnetite alteration. Sample thickness was ≤ 5 mg Fe/cm^2^ to avoid potential saturation effects. Spectra were obtained at room temperature with the spectrometer operating in constant acceleration mode with a velocity ramp of ± 5 mm/s.

For other garnet-bearing samples, the flank method was used to determine the Fe^3+^/ΣFe of garnet with the microprobe following the procedures of Höfer et al.^52^ and Höfer and Brey^53^. Measurement conditions were 15 kV, 120 nA and a beam diameter of 1 µm. Garnet from several samples was analysed by both Mössbauer spectroscopy and the flank method in order to verify consistency between to the two approaches. These yielded the following Fe^3+^/∑Fe ratios: JPN3B Möss = 0.07, flank = 0.08, 0.08, 0.08, 0.10; JPS6B Möss = 0.05, flank = 0.05, 0.03; X04 Möss = 0.03, flank = 0.03, 0.04, 0.02, with multiple flank measurements determined on different grains. The Fe^3+^/ΣFe of spinel from many of the spinel peridotites was determined using the microprobe and employing a set of secondary standards following the measurement protocol of Wood and Virgo^54^. Uncertainties in Fe^3+^/∑Fe are assessed as: ± 0.01 for the determinations by Mössbauer spectroscopy, ± 0.01–0.02 for garnets analysed by the flank method and ± 0.025 for spinels analysed by microprobe using secondary standards^55,56^. Measured Fe^3+^/∑Fe for garnet and spinel are provided in Supplementary Tables 4 and 5.

### Thermobarometry

Equilibration temperatures for the spinel peridotites were calculated using the olivine-spinel equilibrium as calibrated by Li et al.^57^. For one sample from Pelly Bay (TU18), the temperature was estimated from the clinopyroxene geothermometer of Nimis and Taylor^58^ due to the fact that both olivine and orthopyroxene had been destroyed by hydrothermal alteration. The corresponding equilibration pressure for the spinel peridotites was estimated by projecting the calculated temperatures onto the well-established geotherms for Somerset Island^59^ and Pelly Bay^9^. For the garnet-bearing peridotites of Somerset Island, temperatures and pressures were calculated iteratively using the olivine-garnet geothermometer of O’Neill and Wood^60^ in combination with the garnet-orthopyroxene geobarometer of Brey and Köhler^61^. Uncertainties in pressure are considered to be about ± 0.3 GPa^61^. For the Pelly Bay suite, the alteration of olivine and orthopyroxene in samples TU15 and TU16 necessitated the application of the Ni-in-garnet geothermometer^62^. Considering the similarities in major and trace element composition of garnet between samples TU15 and TU14-b2 and between TU16 and TU13-a2-3 (see Supplementary Tables 4, 6), the orthopyroxene composition from the latter samples was applied to TU15 and TU16 in order to estimate a pressure of equilibration using the garnet-orthopyroxene geobarometer^61^. To maintain consistency within this sample suite, the temperature of all four garnet-bearing samples from Pelly Bay were computed on the basis of the Ni-in-garnet geothermometer^62^. The estimates of temperature and pressure of equilibration for all samples are listed in Supplementary Table 7.

### Calculation of oxygen fugacity

Combining measured Fe^3+^/∑Fe for garnet and spinel as described above with their respective total Fe contents determined from electron microprobe analysis, we can derive Fe^3+^ concentrations in these two phases. This information, along with the results of thermobarometry (Supplementary Table 7) enables calculation of the ƒO_2_ conditions experienced by each sample prior to entrainment using equilibria involving the Fe-bearing components of olivine, orthopyroxene and either garnet or spinel. The ƒO_2_ values, expressed as ∆logƒO_2_ relative to the fayalite–magnetite–quartz reference oxygen buffer (FMQ) were computed from the oxybarometer calibrations of Stagno et al.^30^ for garnet-bearing and Wood et al.^31^ for spinel-bearing peridotites (see Supplementary Table 7). For five samples that contained garnet and spinel, we were able to estimate ∆logƒO_2_ values based upon both equilibria (Supplementary Table 7). Uncertainties in the ƒO_2_ calculations are estimated to be ± 0.6 and ± 0.5 log units for the garnet and spinel-based calibrations, respectively. Since both olivine and orthopyroxene were strongly altered in samples TU15, TU16 and TU18, we estimated the ƒO_2_ using olivine-orthopyroxene pairs borrowed from other samples that had similar compositional characteristics. For TU18, we made calculations with the silicate compositions from K13A1, K13A4 and K15a4 since the cr# of spinel in these three samples (0.34, 0.25, 0.35, respectively) effectively brackets that measured in TU18 (0.31). The difference in ∆logƒO_2_ calculated from these three different olivine-orthopyroxene pairs was ~ 0.1 log units; much smaller than the overall uncertainty of the calibration. The silicates from TU13-a2-3 and TU14-b2 were respectively used for samples TU16 and TU 15, since these samples contain garnet with very similar compositions, including trace element signatures and derived Ni-in-garnet temperatures (see Supplementary Tables 4, 6 and 7).

## Supplementary Information

Supplementary Figures.

Supplementary Table 1.

Supplementary Table 2.

Supplementary Table 3.

Supplementary Table 4.

Supplementary Table 5.

Supplementary Table 6.

Supplementary Table 7.
