# Supplementary material for: Oxidation state and metasomatism of the lithospheric mantle beneath the Rae Craton, Canada: strong gradients reflect craton formation and evolution
Source: Sci Rep. 2021 Feb 11;11:3684. doi: 10.1038/s41598-021-83261-6 (PMC7878471; doi:10.1038/s41598-021-83261-6)
Supplement: Supplementary file 1 — Supplementary Figures. [file 41598_2021_83261_MOESM1_ESM.pdf]

Supplementary Information

Oxidation state and metasomatism of the lithospheric mantle beneath the Rae Craton, Canada – implications for craton formation and evolution

Alan B. Woodland[1,2], Carolin Gräf[1], Theresa Sandner[1], Heidi E. Höfer[1], Hans-Michael Seitz[1,2], D. Graham Pearson[3], Bruce A. Kjarsgaard[4]

[1]Institut für Geowissenschaften, Goethe-Universität Frankfurt, Altenhöferallee 1, 60438 Frankfurt am Main, Germany
[2]Frankfurt Isotope and Element Research Center (FIERCE), Goethe-Universität Frankfurt, Frankfurt am Main, Germany
[3]Department of Earth and Atmospheric Sciences, University of Alberta, 1-26 Earth Science Building, Edmonton, Alberta, Canada T6G 2E3
[4]Geological Survey of Canada, 615 Booth Street, Ottawa, Ontario, Canada K1A 0E9

This file includes:

Supplementary Figure S1 with Text: Textures of peridotite samples

Supplementary Figure S2: Plot of Al_2O_3 (in wt %) versus molar Mg# for clinopyroxene

Supplementary Figure S3: Plot of Cr_2O_3 versus CaO (wt %) for garnet

Supplementary Figure S4: Variation in Cr# and Mg# in Spinels

Supplementary Figure S5: Trace element concentrations in garnet as a function of depth

Supplementary Figure S6 with Text: Calculated garnet-clinopyroxene partition coefficients

Textures of peridotite samples:

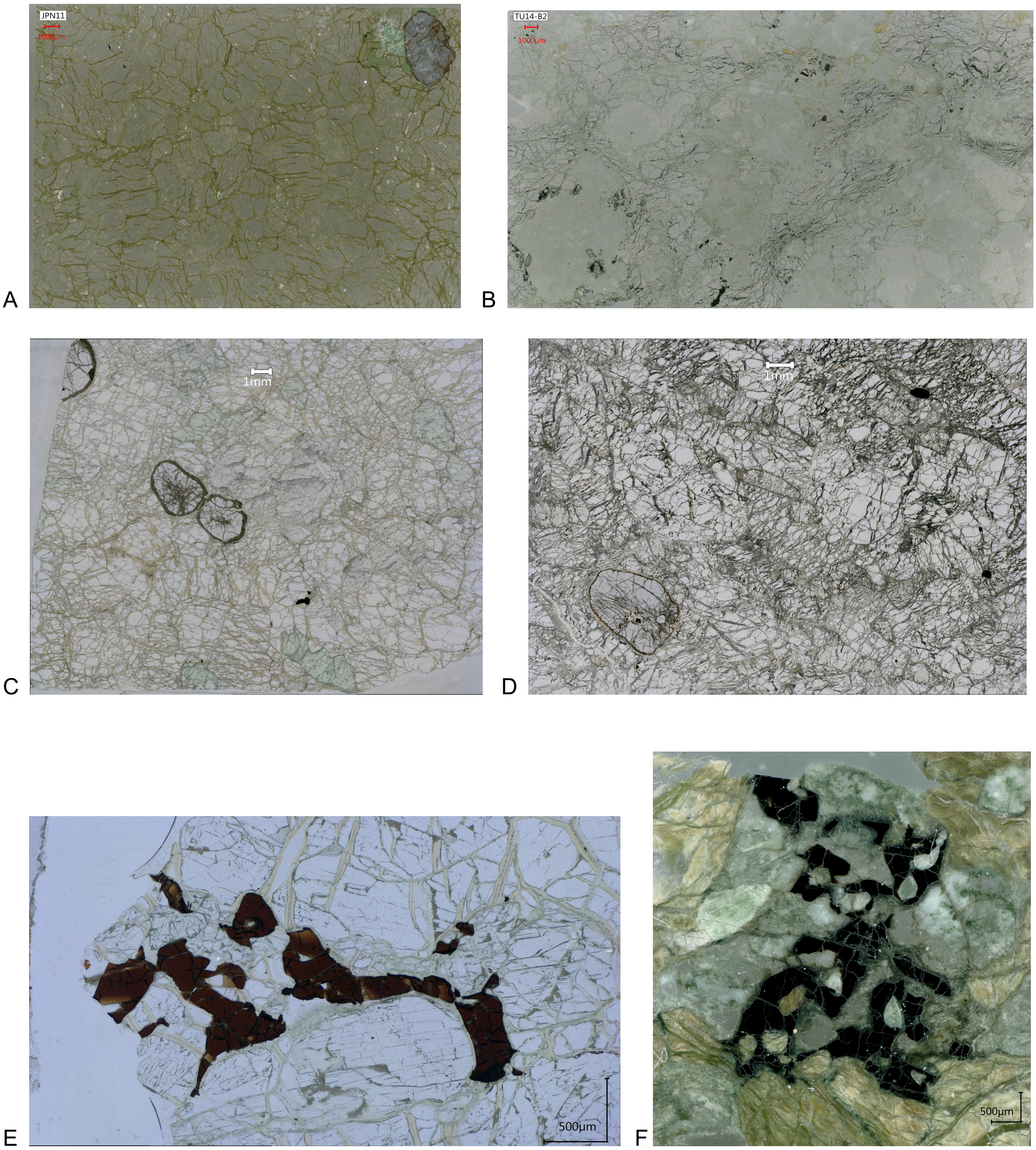

Figure S1. Examples of rock textures from the studied suite of peridotite xenoliths from Somerset Island and Pelly Bay: A) course equal to tabular texture of sample JPN11, B) porphyroclastic texture of sample TU14, C) and D) examples of garnet-spinel peridotite samples JP1-X2 and JPS1, respectively, illustrating that the spinel (small dark grains) is often not in direct contact with garnet (pinkish grains with high relief), E) and F) Vermicular spinel texture in samples K13-A3 and TU18, respectively.

Further petrologic descriptions of the investigated samples from Somerset Island and Pelly Bay are provided in Irvine et al.[17] and Lui et al.[9], respectively.

Mineral compositions:

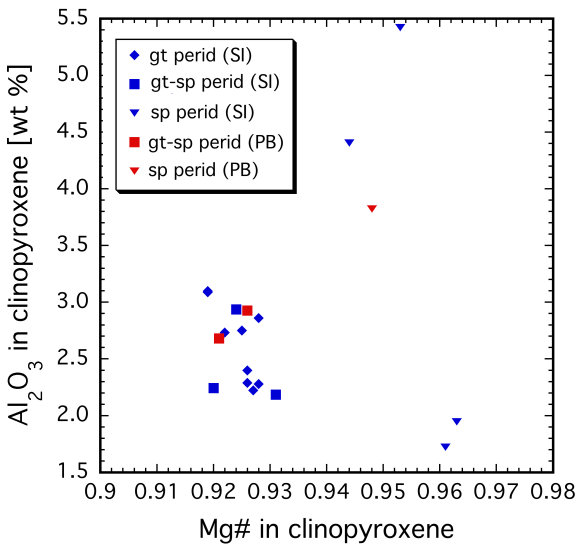

Figure S2. Plot of Al_2O_3 (in wt %) versus molar Mg# for clinopyroxene from Somerset Island (SI) and Pelly Bay (PB).

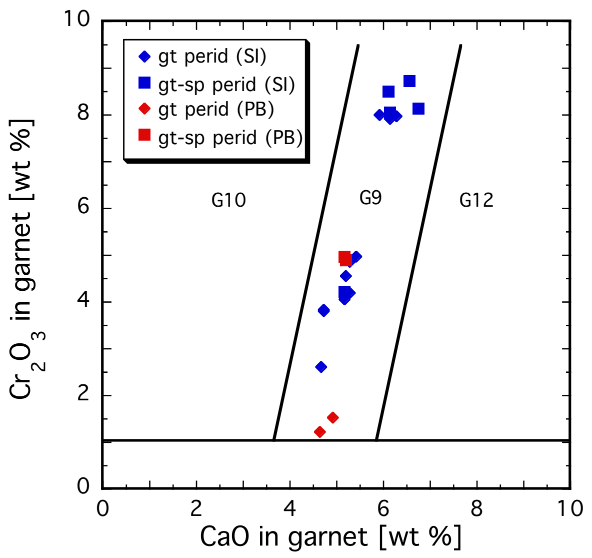

Figure S3. Plot of Cr_2O_3 versus CaO (wt %) for garnet. Classification boundaries are from Grütter et al.[25]. All garnets plot within the G9, lherzolite field.

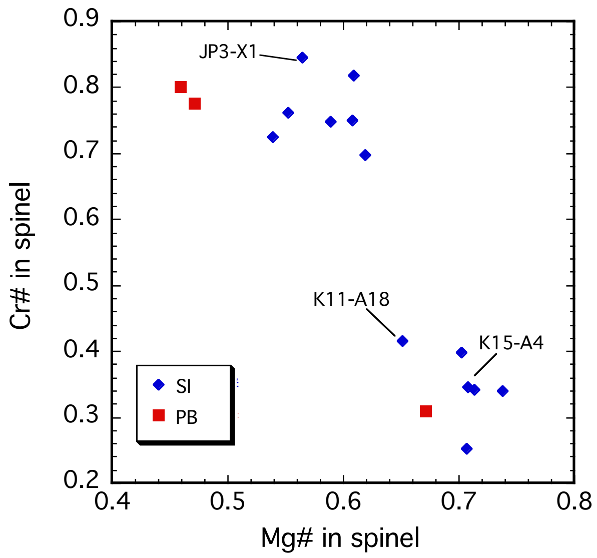

Figure S4. Variation in Cr# and Mg# in Spinels

Trace element concentrations in garnet as a function of depth:

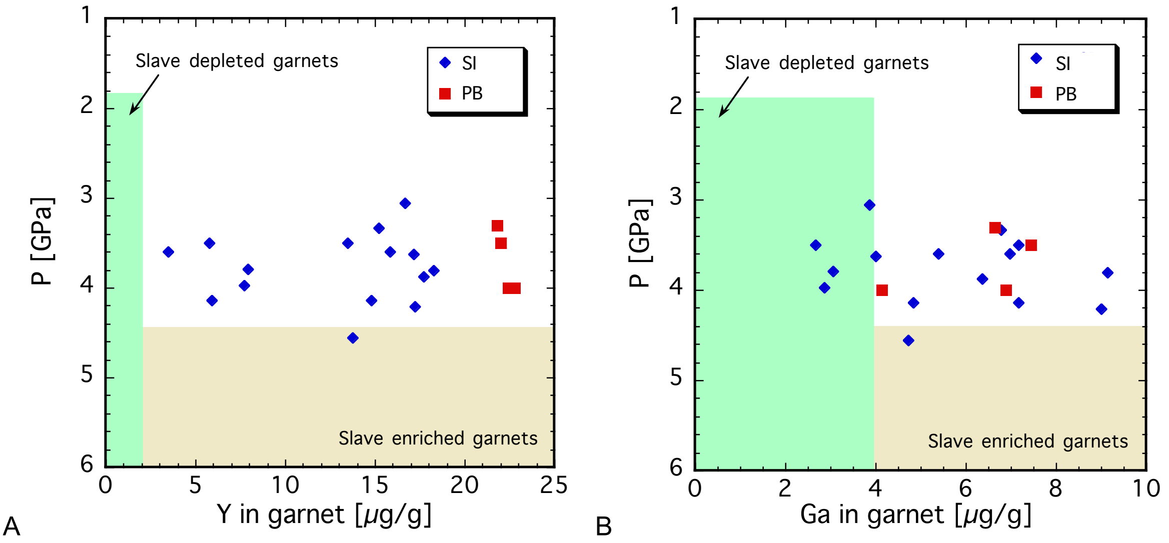

Figure S5. Variation in A) Y and B) Ga contents of garnet plotted as a function of calculated depth. The shaded fields are for depleted and enriched garnets from the Slave craton as reported by Yaxley et al.[42].

Calculated garnet-clinopyroxene partition coefficients

Partition coefficients ($D^{gt/cpx}$) computed from our data (Supplementary Table 6) are compared with equilibrium values reported by Zack et al.[36] in Figures S6A and S6B. $D^{gt/cpx}$ values derived from most samples exhibit the same systematics as those of Zack et al.[36], even if some samples are shifted to somewhat higher or lower values.

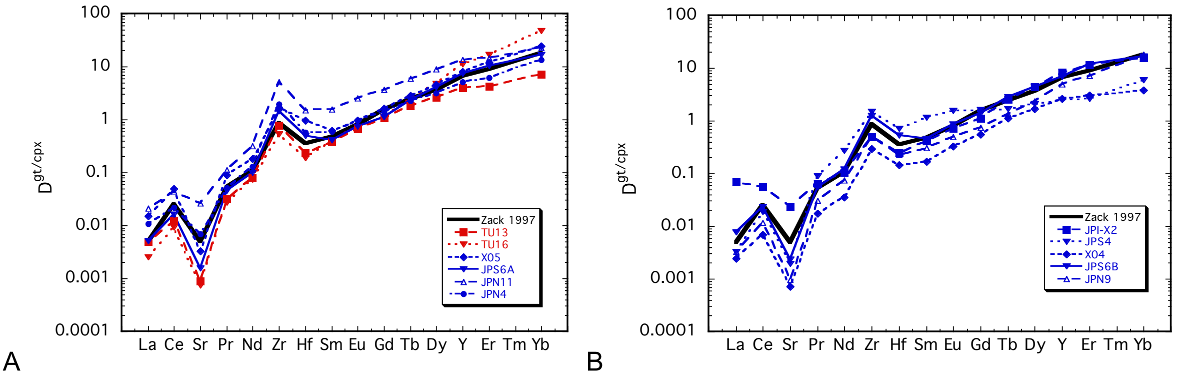

Figure S6. Trace element partitioning behavior between garnet and clinopyroxene compared with equilibrium values reported by Zack et al.[36].
